# “Zombielike” Aggression in Perampanel Overdose

**DOI:** 10.7759/cureus.14971

**Published:** 2021-05-11

**Authors:** Rami Z Morsi, Jeffrey A Katz

**Affiliations:** 1 Neurology, University of Chicago, Chicago, USA; 2 Anesthesia, NorthShore University HealthSystem, Evanston, USA

**Keywords:** perampanel, antiepileptic drug, epilepsy, suicide attempt, adverse effect

## Abstract

Perampanel is an antiepileptic drug that blocks amino-3-hydroxy-5-methyl-4-isoxazolepropionic acid-type glutamate receptors. To date, little is known about the management of perampanel intoxication. We report a case of “zombielike” behavior secondary to intentional perampanel overdose. An 18-year-old male with idiopathic generalized epilepsy and focal features presented with aggressive and “zombielike” behavior after suicide attempt via intentional perampanel overdose, amounting to approximately 128 mg. Clinical symptoms gradually improved with continuous dexmedetomidine infusion and intravenous lorazepam boluses five days after being admitted to the intensive care unit. While perampanel intoxication has been reported to be associated with central nervous system-related adverse effects, awareness of this association is necessary to prompt more appropriate management tailored to perampanel toxicity.

## Introduction

Perampanel is the first antiepileptic drug in its class to be approved by the Food and Drug Administration for its adjunctive treatment in patients with epilepsy aged 12 years or older, more specifically in those with partial-onset seizures with or without secondary generalization [1]. Perampanel noncompetitively blocks amino-3-hydroxy-5-methyl-4-isoxazolepropionic acid-type glutamate receptors on post-synaptic neurons to reduce excitatory neurotransmission and spread of seizure activity [2,3].

While Phase III clinical studies demonstrate perampanel to be safe and well tolerated with continued effectiveness in improving seizure control, dose-dependent central nervous system (CNS) adverse effects have been reported. These dose-dependent treatment-emergent adverse events (TEAEs) include, but are not limited to, dizziness, fatigue, and somnolence [4]. In a pooled analysis of psychiatric and behavioral safety data conducted by Ettinger and colleagues, the most common dose-dependent TEAEs were irritability followed by aggression with overall rates of psychiatric TEAEs being higher in perampanel groups compared to placebo groups [5]. The FYDATA study conducted by Villanueva and colleagues found that patients treated with perampanel, particularly those with prior psychiatric comorbidities, such as hyperactivity and personality disorders, were more likely to experience adverse effects, such as dizziness, somnolence, and irritability, at 12 months [6]. Another study by Lee and colleagues demonstrated that pre-existing depression and a perampanel dose 8 mg daily were potential predictors for aggression. Interestingly, however, they also found that topiramate, when used in conjunction with perampanel, was protective against aggression in patients with focal epilepsy [7].

To date, little is known about the management of perampanel intoxication. Hoppner and colleagues report a suicide attempt of a 34-year-old female patient with symptomatic focal epilepsy due to tuberous sclerosis who ingested 204 mg of perampanel, which resulted in impaired consciousness and delirium, and was admitted for close monitoring [8]. Wu and colleagues also describe a case of a 40-year-old male patient with prior psychiatric comorbidities and no history of seizure disorder who ingested 60 mg of perampanel in a suicide attempt, resulting in severe aggression and was managed with haloperidol, lorazepam, and dexmedetomidine [9]. Here, we describe the clinical findings in a patient with history of idiopathic generalized epilepsy with focal features who ingested an estimated 128 mg of perampanel in a suicide attempt and presented with bizarre, aggressive, and “zombielike” behavior.

## Case presentation

The patient was an 18-year-old African American male with a history of idiopathic generalized epilepsy with focal features, history of medication noncompliance, no documented formal psychiatric comorbidities, history of substance use, and family history of seizures, who presented after suicide attempt via intentional overdose of perampanel. Patient started taking 8 mg of perampanel daily for 15 months prior to presentation. Patient had multiple trials of antiepileptic medications in the past, including levetiracetam, valproic acid, and lacosamide but patient continued to have breakthrough seizures despite dosage adjustments. On the day of presentation, after a psychosocial stressor provoking the patient to damage the house television, patient ingested his prescription of perampanel, estimated to be 128 mg, which was 16 times his daily dose. Patient was brought into the emergency department via emergency medical services after patient’s mother found him on the floor of his bedroom unresponsive, followed by agitation, paranoia about family members’ remarks about his actions, and physically aggressive behavior. On arrival in the emergency department, his temperature was 97.3^o^F (36.3^o^C), blood pressure was 142/88 mm Hg, pulse was 78 beats/min, respiratory rate was 18 breaths/min, and oxygen saturation (SpO2) was 100% on room air. On physical examination, patient was awake and remained agitated. Upon further physical examination, patient was noted to have pupils equal, round, and reactive to light, moist mucous membranes, no increased work of breathing with symmetric chest rise, and warm and dry skin. Patient was moving all extremities in an erratic jerking motion and displaying overtly aggressive and physically harmful behavior toward staff including grabbing and punching anyone in close proximity. Initial laboratory testing was only significant for mild leukocytosis of 11.4*10^3^/µL (normal range: 4.0-10.0*10^3^/µL). Additional testing including serum electrolytes, hepatic function, creatine kinase, blood alcohol level, salicylate level, acetaminophen level, and head computed tomography scan were all negative for any acute abnormalities. Electrocardiogram (EKG) also showed no acute abnormalities, with a corrected QT (QTc) interval of 420 milliseconds. Due to combative and aggressive behavior, patient was placed in four-point restraints, given 0.5 mg of lorazepam, and admitted to the intensive care unit (ICU) for close monitoring and further management. Poison control was contacted for additional assistance in management. 

Overnight, patient received a total of four IV boluses of 0.5 mg of lorazepam after increased anger and aggression requiring three staff members to restrain patient for continued violent behavior including thrashing on his bed, kicking, punching, and biting staff members. One staff member had a severe bite wound that required medical intervention in the emergency department. Haloperidol was not initially given at the time for fear of interaction with perampanel, and dexmedetomidine was not given for lack of evidence regarding perampanel intoxication. During the first hospital day, patient was still extremely agitated after requiring a total of 11 mg of IV lorazepam and 5 mg of IV haloperidol throughout the day. His vital signs were within normal limits during this time, but patient had extreme liability between somnolence with blank staring at ceiling followed by severe agitation and violence toward self and others if any person was in close proximity. 

On Day 2 of hospitalization, patient continued to display this labile behavior despite receiving a total of 10 mg of IV lorazepam boluses. Due to the lability of his behavior and the ineffectiveness of lorazepam, he was started on a continuous infusion of dexmedetomidine titrated to 0.9 mcg/kg/h coupled with IV lorazepam boluses amounting to a total of 6 mg after further discussion with the poison control service. On Day 3 of hospitalization, patient’s agitation was controlled with a continuous dexmedetomidine infusion at a rate of 0.7 mcg/kg/h combined with a total of 6 mg of IV lorazepam. On Day 4 of hospitalization, patient remained on continuous dexmedetomidine infusion at a rate of 0.6 mcg/kg/h combined with a total of 8 mg of IV lorazepam. Over the course of Day 5 of hospitalization, the continuous dexmedetomidine infusion was weaned off, and patient only required 2 mg of IV lorazepam. Patient was more awake, oriented, calm, and cooperative. Due to his improvement in mental status and no longer requiring sedation, patient was transferred out of the ICU. Patient did not require any additional sedative medications while on general medicine floors. His blood pressure was 138/83 mm Hg, heart rate was 81 beats/min, respiratory rate was 18 breaths/min, and SpO2 was 98% on room air. During his time on general medicine floors, repeat EKG showed a prolonged QTc of 460 milliseconds but patient continued to be well oriented, calm, and cooperative, and was transferred to psychiatric inpatient services voluntarily. Patient was started on 100 mg of zonisamide daily after discharge and gradually increased to 200 mg daily and has had no seizures or changes in mood or behavior since. 

## Discussion

To the best of our knowledge, this is the first case report of a patient with a documented epilepsy disorder presenting with severe agitation and aggression mimicking zombielike behavior after perampanel overdose who was treated with continuous dexmedetomidine infusion and IV boluses of lorazepam. In New York, synthetic cannabinoid methyl 2-(1-(4-fluorobenzyl)-1H-indazole-3-carboxamido)-3-methylbutanoate (AMB-FUBINACA) was associated with a “zombie” outbreak consisting of “zombielike” groaning, slow limb movement, blank stares, and delayed response to questioning [10], but this is the first case report illustrating “zombielike” behavior consisting of moaning, blank stares, erratic limb movement, grabbing, and biting actions associated with perampanel overdose. Revonsuo and colleagues highlight that “Zombiehood” implies automatic behaviors -- whether routine or nonroutine, such as open eyes in a “glazed” appearance, detachment from the environment, bizarre behaviors encompassing aggressive and defensive measures -- can be carried out, even in a state of impaired consciousness [11]. Perampanel has been associated with several psychiatric adverse events as evidently described by Ettinger and colleagues, but there is no clear management plan for perampanel overdose [5]. Wu and colleagues reported a case of a suicide attempt in an individual with prior psychiatric comorbidities but no documented epilepsy and were able to control the patient’s agitation using continuous infusions of dexmedetomidine and haloperidol. This patient described by Wu and colleagues demonstrated improvement in mental status after four days of hospitalization, which was similar to the patient described in this report who needed nearly the same amount of days for the perampanel overdose to gradually clear out of the system [9]. The rationale behind the use of dexmedetomidine was because of its ability to reduce the duration of delirium, agitation, and the need for additional sedation in the ICU setting [12]. Hoppner and colleagues also described a clinical scenario where perampanel overdose did not cause any significant changes in vital signs, such as blood pressure and heart rate, which was similarly seen in the clinical scenario described in this report. However, no clear management plan was described by Hoppner and colleagues [8]. Parsons and colleagues described a case of a 20-year-old treatment-naïve male patient with no prior medical problems who experienced prolonged unconsciousness as a result of a mixed overdose involving perampanel, levothyroxine, and pregabalin [13]. This patient required endotracheal intubation for airway protection, sedation with propofol and fentanyl, and general supportive care [13]. One case was reported by Kim and colleagues regarding a 39-year-old female with documented seizures who experienced altered consciousness after ingesting 40 mg of perampanel and 10,000 mg of valproic acid [14]. This female patient’s hospital stay was further complicated by pulmonary embolism requiring heparin anticoagulation and mechanical ventilation. However, no sedation measures were highlighted in this case study [14].

The long mean half-life of 105 hours and the dose-dependent CNS adverse effects associated with perampanel should be given extreme caution given the negative consequences this drug has on an individual’s mental status when consumed in supratherapeutic levels [15]. Gidal and colleagues demonstrate that while higher plasma concentrations of perampanel are inversely related to seizure frequency, higher perampanel plasma concentrations are, nevertheless, directly related to adverse effects [16]. While clinical trials have demonstrated that common adverse effects of perampanel include somnolence, fatigue, irritability, headache, dizziness, irritability, ataxia, dysarthria, and memory impairment, there have been increasing cases of suicidality related to perampanel intake [5,17]. Perampanel’s ability to penetrate the blood-brain barrier when ingested orally largely attributes to these CNS adverse effects [18]. It has also been hypothesized that perampanel’s AMPA antagonist properties can alter glutamate levels, and, subsequently, lead to aggressive behavior [19,20].

## Conclusions

This case report highlights the need for additional data to understand the pharmacologic effects of perampanel on the CNS as well as uncover mechanisms for managing CNS-related adverse effects induced by perampanel. While continuous dexmedetomidine infusion and lorazepam boluses were considered in this patient’s clinical course, more information is needed to guide medical decisions given the scarcity of the literature on perampanel toxicity-related suicidality and aggression.
